# Machine learning based differentiation of glioblastoma from brain metastasis using MRI derived radiomics

**DOI:** 10.1038/s41598-021-90032-w

**Published:** 2021-05-18

**Authors:** Sarv Priya, Yanan Liu, Caitlin Ward, Nam H. Le, Neetu Soni, Ravishankar Pillenahalli Maheshwarappa, Varun Monga, Honghai Zhang, Milan Sonka, Girish Bathla

**Affiliations:** 1 Department of Radiology, University of Iowa Hospital and Clinics, 200 Hawkins Drive, Iowa City, IA 52242 USA; 2 College of Engineering, University of Iowa, Iowa City, IA USA; 3 Department of Biostatistics, University of Iowa, Iowa City, IA USA; 4 Department of Medicine, University of Iowa Hospitals and Clinics, Iowa City, IA USA

**Keywords:** Cancer imaging, CNS cancer

## Abstract

Few studies have addressed radiomics based differentiation of Glioblastoma (GBM) and intracranial metastatic disease (IMD). However, the effect of different tumor masks, comparison of single versus multiparametric MRI (mp-MRI) or select combination of sequences remains undefined. We cross-compared multiple radiomics based machine learning (ML) models using mp-MRI to determine optimized configurations. Our retrospective study included 60 GBM and 60 IMD patients. Forty-five combinations of ML models and feature reduction strategies were assessed for features extracted from whole tumor and edema masks using mp-MRI [T1W, T2W, T1-contrast enhanced (T1-CE), ADC, FLAIR], individual MRI sequences and combined T1-CE and FLAIR sequences. Model performance was assessed using receiver operating characteristic curve. For mp-MRI, the best model was LASSO model fit using full feature set (AUC 0.953). FLAIR was the best individual sequence (LASSO-full feature set, AUC 0.951). For combined T1-CE/FLAIR sequence, adaBoost-full feature set was the best performer (AUC 0.951). No significant difference was seen between top models across all scenarios, including models using FLAIR only, mp-MRI and combined T1-CE/FLAIR sequence. Top features were extracted from both the whole tumor and edema masks. Shape sphericity is an important discriminating feature.

## Introduction

Glioblastoma (GBM) and intracranial metastatic disease (IMD) together constitute the vast majority of malignant brain neoplasms^1,2^. Gliomas account for about 25.5% of all primary brain and other CNS tumors and approximately 80.8% of primary malignant brain tumors. Of these, GBM is the most common, accounting for over half of the gliomas (57.3%) with an annual age-adjusted incidence rate of 3.22 per 100,000 population in the United States^3^. IMD on the other hand has an incidence rate of approximately 10 per 100,000 population and are more common than GBM^1^.

The distinction between GBM and IMD is important since it has diagnostic, therapeutic and prognostic implications^2,4,5^. Histopathological tissue confirmation is considered the gold standard for diagnosis, but is not always optimal, with misdiagnosis and under grading of tumors reported in 9.2 and 28% of lesions respectively^6^. The reported biopsy complication rate varies between 6 and 12% with mortality rate of 0–1.7%^7^.

On conventional imaging, factors such as multiplicity of lesions, morphology, cerebellar localization and known history of underlying primary cancer can be helpful to differentiate IMD from GBM^1,2^. However, brain metastases may present as a solitary lesion in approximately half of the patients or be associated with undiagnosed systemic malignancy in about 15–30%^8,9^. Thus, conventional imaging alone may be insufficient for accurate classification. Prior studies using advanced MRI imaging techniques such as perfusion imaging^10–12^, spectroscopy, diffusion-weighted and tensor imaging^13^, new diffusion weighted techniques like neurite orientation dispersion and density imaging^14^, and more recently other advanced sequences like non-contrast inflow-based vascular-space-occupancy MR imaging^15^ have been used to distinguish amongst these entities with variable success^16–20^. However, these advanced imaging sequences are not performed universally, and conventional imaging is still the mainstay in clinical practice. Radiomics is a technique applied on medical images to extract quantitative features invisible to human eye^21^. These features may provide a complimentary tool for the expert human reader. These radiomic features have been employed in multiple prior studies for tumor grading, classification and prognosis^21–24^. The advantage of radiomics is that it can be applied to routinely acquired conventional clinical images^25^.

The application of radiomics based machine learning techniques (MLT) to differentiate GBM from IMD has only been explored in a few prior studies, mostly using limited MRI sequences and MLT^1,2,4,26–29^. The superiority of having one, a few, or all conventional MRI sequences (T1 WI, T2 WI, ADC, FLAIR and T1-CE) as well as the impact of feature reduction and type of machine learning models remain largely unexplored. In this study, we aimed to determine the optimal radiomics based MLT for this specific two-class problem using routinely available conventional MRI sequences.

## Results

### Patient characteristics

There were 120 patients (males 63, females 57) in the study population (GBM 60, metastases 60). The majority of metastatic tumors were from lung cancer (40) followed by breast cancer (20). The demographic and tumor characteristics are provided in Table 1.

**Table 1:** Patient demographics and tumor characteristics.

	GBM	METASTASES
Patients (120)	60	60; Breast (20); Lung (40)
Age years (mean ± SD)	62 ± 11	62 ± 10
**Gender**		
Male	36	27
Female	24	33
**Localization**		
Supratentorial	58	Breast (10); Lung (25)
Infratentorial	2	Breast (6); Lung (8)
Both	0	Breast (4); Lung (7)
**Multiplicity**		
Single	53	Breast (12); Lung (24)
Two	5	Breast (2); Lung (7)
≥ Two (Multiple)	2	Breast (6); Lung (9)
**Necrosis**		
Yes	59	Breast (12); Lung (24)
No	1	Breast (8); Lung (16)

GBM-Glioblastoma.

### Model performance on mp-MRI

**Figure 1:**
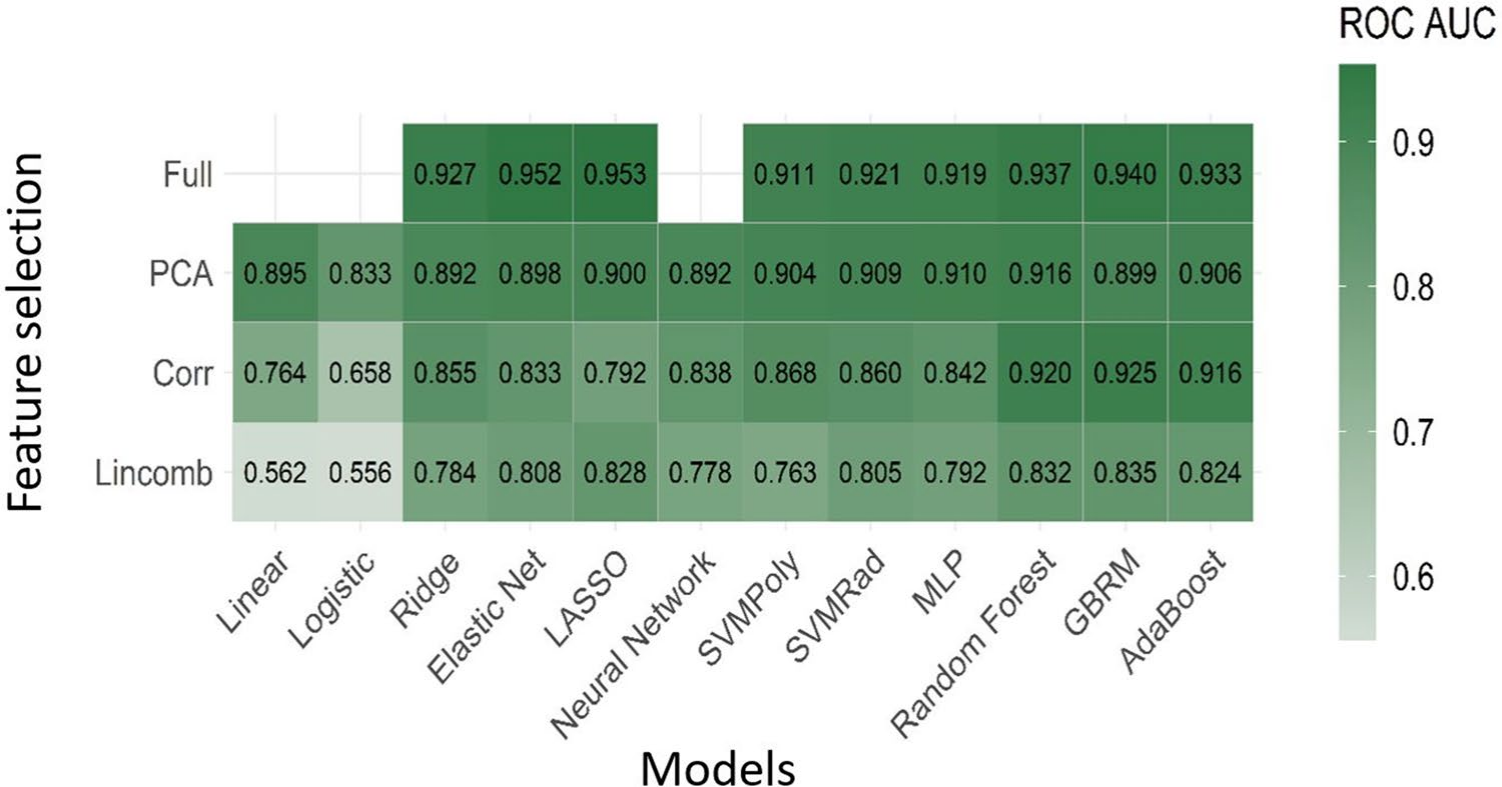
Diagnostic performance using multiparametric MRI. Mean cross-validated ROC AUC for all 45 machine learning and feature reduction combinations using all sequences.

Using mp-MRI, the two best performing models were the LASSO (least absolute shrinkage and selection operator) and elastic net fit to the full feature set. The LASSO classifier had mean cross-validated area under the curve (AUC) of 0.953 and the elastic net classifier had a mean cross-validated AUC of 0.952. Figure 1 displays the mean cross-validated AUC for all 45 MLT combinations fit using all sequences.

### Model performance on individual sequences

**Figure 2:**
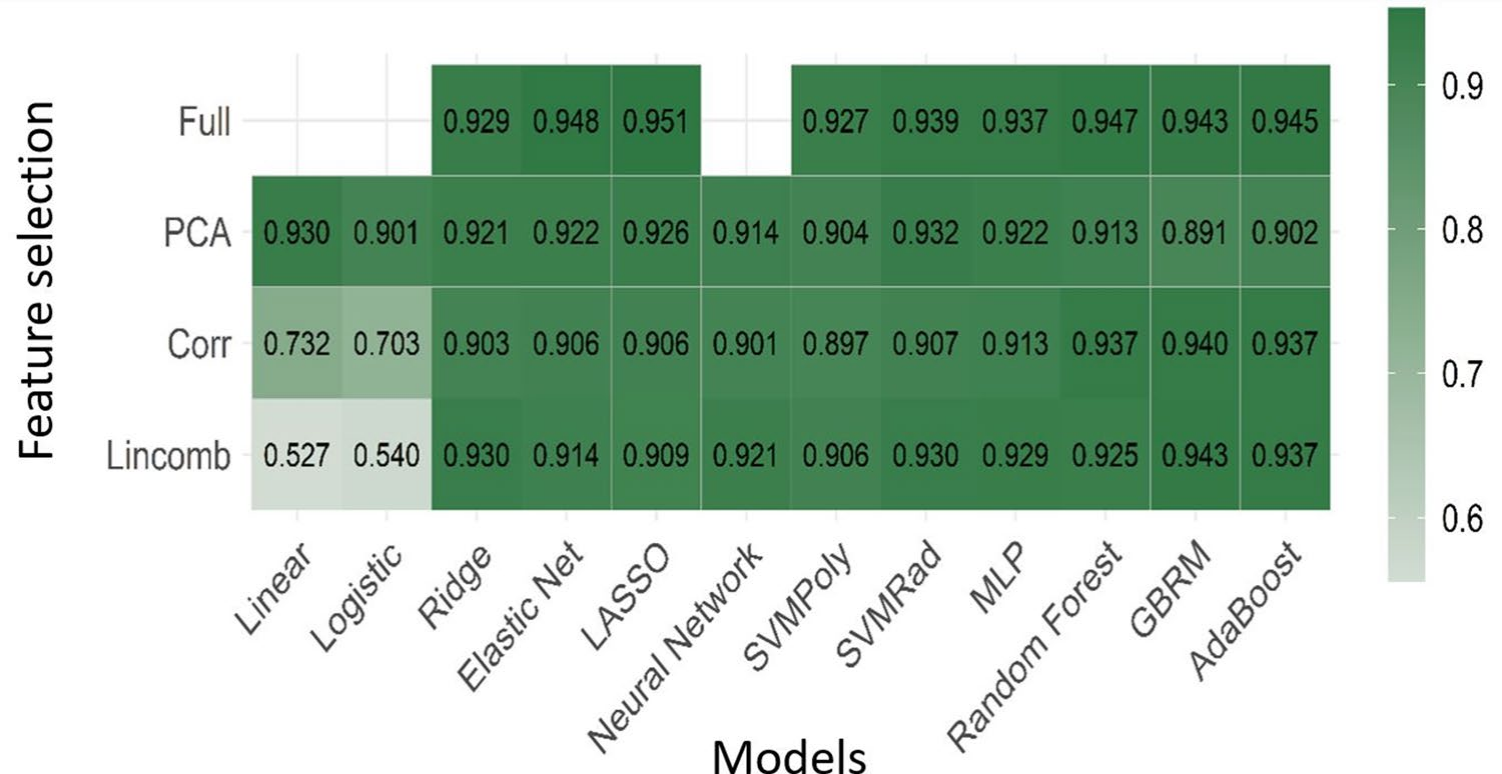
Diagnostic performance using FLAIR sequence. Mean AUC for all models fit using the FLAIR sequence as many of the top performing models came from this sequence.

For the models fit to each sequence separately, the LASSO and elastic net fit to the full feature set again were the top performing models, with both being fit to the FLAIR sequence. Interestingly, seven of the top 10 best performing sequence-specific models were derived from the FLAIR sequence. The LASSO classifier on the FLAIR sequence had mean cross-validated AUC of 0.951 while the elastic net classifier had a mean cross-validated AUC of 0.948. Figure 2 shows the mean AUC for all models fit using the FLAIR sequence as many of the top performing individual sequence models came from this sequence. Table 2 displays the mean and standard deviation of AUC for the 10 best performing models for mp-MRI and individual sequences.

**Table 2:** Top ten best performing models using all sequence and sequence-specific features sets ordered by ROC AUC.

All sequences			Sequence-specific			
Model	Feature reduction	ROC AUC mean (SD)	Model	Feature reduction	Sequence	ROC AUC mean (SD)
Lasso	Full	0.953 (0.041)	Lasso	Full	F	0.951 (0.049)
Enet	Full	0.952 (0.038)	Enet	Full	F	0.948 (0.049)
GBRM	Full	0.940 (0.040)	RF	Full	F	0.947 (0.042)
RF	Full	0.937 (0.046)	ada	Full	F	0.945 (0.038)
ada	Full	0.933 (0.045)	Lasso	Full	CE	0.943 (0.041)
Ridge	Full	0.928 (0.053)	GBRM	Full	F	0.943 (0.045)
GBRM	Corr	0.925 (0.056)	GBRM	Lincomb	F	0.943 (0.045)
SVMRad	Full	0.921 (0.042)	Lasso	Full	T1	0.941 (0.046)
RF	Corr	0.920 (0.045)	Enet	Full	T1	0.941 (0.041)
MLP	Full	0.919 (0.055)	GBRM	Corr	F	0.940 (0.046)

Enet: elastic net; Lasso (least absolute shrinkage and selection operator; GBRM: generalized boosted regression model; RF: random forest; ada: boosting of classification trees with adaBoost; SVMRad: SVM with a radial kernel; MLP: multi-layer perceptron; full: full feature set; corr: High correlation filter; lincomb: linear combinations filter; F: FLAIR; CE: T1-CE.

### Model performance from combined T1-CE and FLAIR sequences

**Figure 3:**
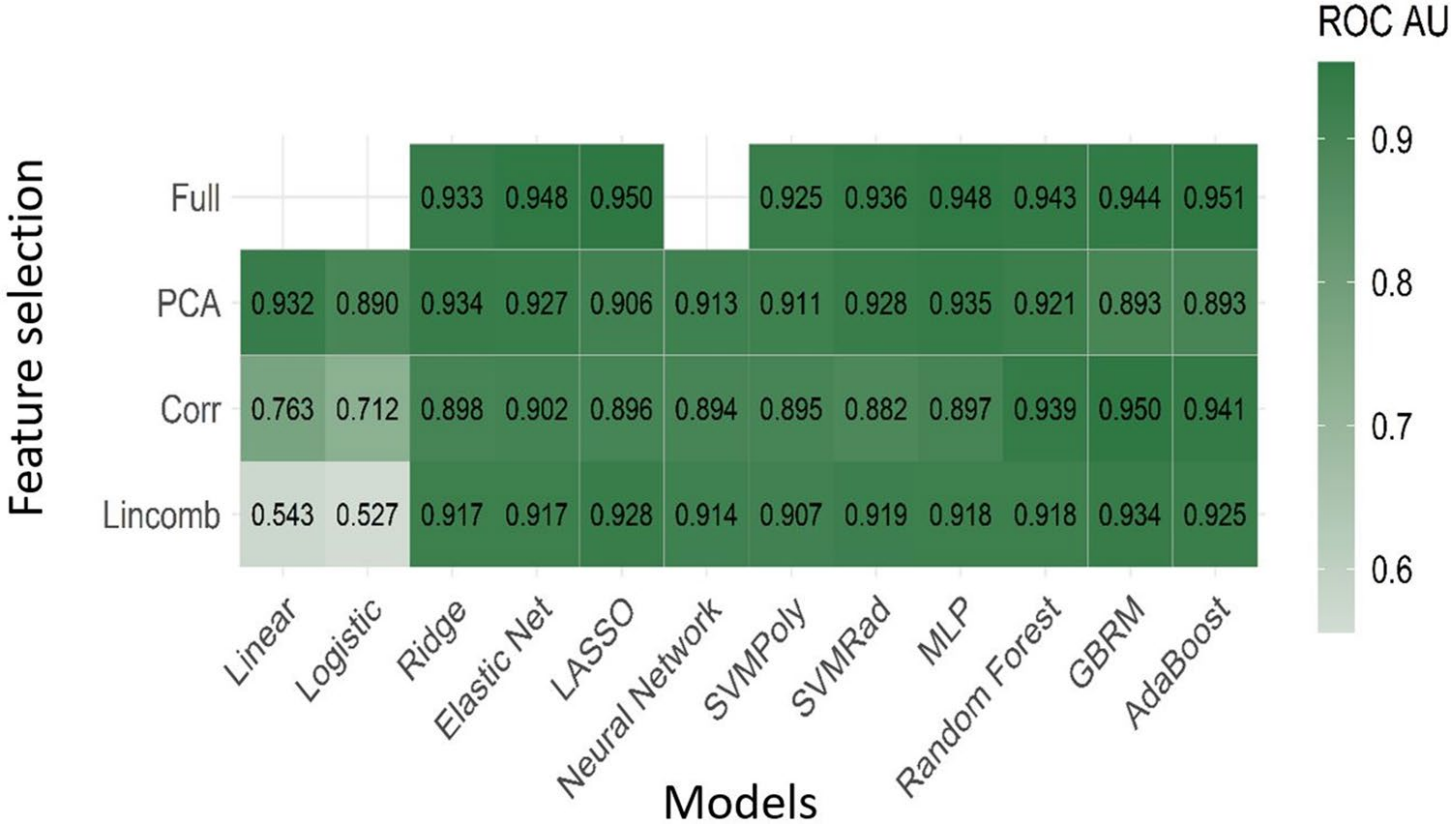
Diagnostic performance using combined T1- contrast enhanced (T1-CE) and FLAIR sequence. Mean AUC for all models fit using the combined T1-CE and FLAIR sequences.

For the models fit to the T1-CE and FLAIR sequences in combination, the adaBoost and LASSO models fit to the full feature set were the top performing models. The adaBoost classifier had mean cross-validated AUC of 0.951. The LASSO classifier had mean cross-validated AUC of 0.950. Figure 3 shows the mean AUC for all models fit using the combined T1-CE and FLAIR sequences. Table 3 displays the mean and standard deviation of AUC for the 10 best performing models for T1-CE and FLAIR combination.

**Table 3:** Top ten best performing models using combination of T1-CE and FLAIR sequences ordered by ROC AUC.

Combination of CE and F sequences		
Model	Feature reduction	ROC AUC mean (SD)
ada	Full	0.951 (0.040)
Lasso	Full	0.950 (0.042)
GBRM	Corr	0.950 (0.040)
Enet	Full	0.948 (0.041)
MLP	Full	0.948 (0.042)
GBRM	Full	0.944 (0.043)
RF	Full	0.943 (0.044)
ada	Corr	0.941 (0.037)
RF	Corr	0.939 (0.039)
SVMRad	Full	0.936 (0.042)

ada: boosting of classification trees with adaBoost; Lasso (least absolute shrinkage and selection operator; GBRM: generalized boosted regression model; Enet: elastic net; MLP: multi-layer perceptron; rf: random forest;; SVMRad: SVM with a radial kernel; full: full feature set; corr: High correlation filter; F: FLAIR; CE: T1-CE.

### Comparison of predictive performance between mp-MRI, individual sequence, and combination of T1-CE with FLAIR

Overall, the best performing model using mp-MRI (LASSO fit to the full feature set), FLAIR sequence (LASSO full) and combined T1-CE and FLAIR sequences (adaBoost full) had similar predictive performance (p- value > 0.05 for all) (Table 4). These results indicate no statistically significant differences in predictive performance between the top models in each of the three scenarios.

**Table 4:** Mean (SD) of performance metrics for two best performing models using all sequences, individual sequences, and combined T1-CE and FLAIR sequences.

Performance metric	Model					
	LASSO full All Seqs	Elastic net full All Seqs	LASSO full F	Elastic net full F	adaBoost full CE + F	LASSO full CE + F
Brier	0.088 (0.036)	0.088 (0.036)	0.083 (0.042)	0.088 (0.043)	0.086 (0.040)	0.102 (0.026)
Accuracy	0.892 (0.061)	0.892 (0.063)	0.897 (0.054)	0.885 (0.054)	0.888 (0.063)	0.887 (0.070)
ROC AUC	0.953 (0.041)	0.952 (0.038)	0.951 (0.049)	0.948 (0.049)	0.951 (0.040)	0.950 (0.042)
Sensitivity	0.887 (0.086)	0.893 (0.092)	0.917 (0.064)	0.903 (0.071)	0.900 (0.080)	0.907 (0.088)
Specificity	0.897 (0.073)	0.890 (0.079)	0.877 (0.094)	0.867 (0.102)	0.877 (0.111)	0.867 (0.115)

LASSO (least absolute shrinkage and selection operator; Enet: elastic net; ada: boosting of classification trees with adaBoost; F: FLAIR; CE: T1-CE.

### Feature importance for the models

**Figure 4:**
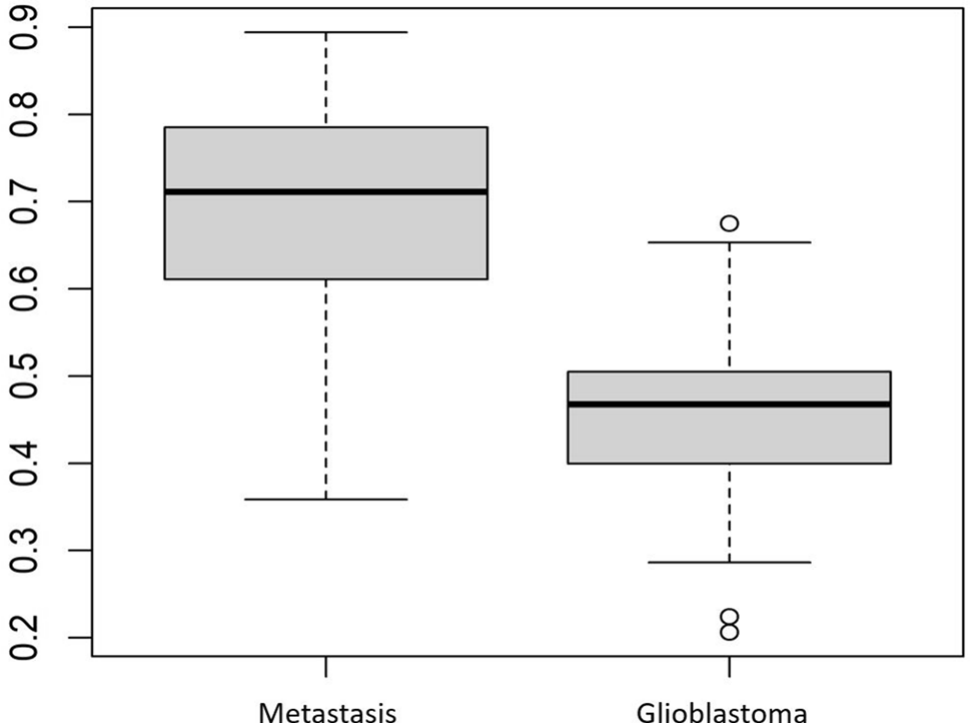
Feature importance. A boxplot showing the distribution of shape sphericity feature for the two tumor types on FLAIR sequence.

Features with higher relative importance were derived from both the whole tumor and edema masks. The shape sphericity was the most important feature in all sequence combinations. A boxplot showing the distribution of this feature for the two tumor types on FLAIR sequence is shown in Fig. 4. Supplementary tables S1-S3 (and supplementary Fig. 1–3) display the ranking by variable importance for the ten most important features for the two best models for mp-MRI, FLAIR and combination of T1-CE and FLAIR sequence. Supplementary Fig. 4 shows the heatmap of feature importance for mp-MRI models (features with relative importance greater than 40 were included).

## Discussion

Our study showed that radiomics based MLT can differentiate GBM and IMD with excellent performance. We found LASSO and elastic net as the top performing models. Another key observation from our study was that the diagnostic performance for best models was similar for mp-MRI, FLAIR sequence and combined T1-CE and FLAIR sequence. Finally, radiomic features with high relative importance were derived from both the whole tumor and edema masks and shape sphericity was the most important feature.

LASSO and elastic net models are both penalized regression models^30^. LASSO model forces the coefficient estimates of the variables with limited contribution to the outcome to be exactly zero. Elastic net is an extension of LASSO and forces those estimates of minor importance variables to be zero or very close to zero. Elastic net model is also robust to extreme correlations among predictors^30,31^. Both of these models help reduce data overfitting. In addition, the nested cross-validation used in our study helps in model hyperparameter optimization and reduce bias in model selection, thereby improving generalizability of the model^32^.

The diagnostic performance for majority of the top performing models in our study for all sequence combinations ranged between AUC 0.936- 0.953. This highlights the fact that albeit small, model performance can vary depending upon the combination of chosen classifier and feature reduction method. Additionally, compared to few prior radiomics studies showing multilayer perceptron (MLP)^26^ and support vector machine (SVM)^27^ as high performing models in GBM and IMD classification we also found MLP (AUC 0.919) and SVM (AUC 0.921) as high performing models. In our study, performance of MLP model was similar (AUC 0.919) compared to a prior study by Ortiz et al^26^ (AUC 0.912). Similarly, SVM model in our study performed superior (AUC 0.921) to a prior study by Qian et al^27^ where performance of SVM model was AUC 0.900). However, both MLP and SVM were not among the highest performing models in our study and we obtained the best result using embedded LASSO model. These observations suggest that model performance depends upon the available data at hand. As such, reliance on a single classifier type approach may be suboptimal and comprehensive model evaluation should be performed.

Our results also compared favorably to other previously reported studies that also evaluated performance of multiple MLT and feature reduction strategies in classification of GBM versus IMD (supplementary table S4). Bae et al^2^ evaluated seven machine learning classifiers and five feature reduction methods based on radiomics features extracted from T1-CE and T2W images and reported an AUC of 0.890 with an accuracy of 83%. Chen et al^33^ evaluated thirty feature reduction and model combinations for their radiomics study and extracted features only from T1-CE sequence with an AUC of 0.80. In contrast, we reported forty-five combinations of feature reduction and classifier MLT and reported a higher AUC of 0.953 and an accuracy of 89%. Our results were better likely due to feature extraction from mp-MRI sequences. Bae et al. also evaluated deep neural network in their study with an AUC of 0.956 and accuracy of 89%^2^ Though, we did not evaluate deep neural network (DNN) due to its computational complexity, our results were comparable to the study by Bae et al. and suggest that mp-MRI, or even just FLAIR/ FLAIR with T1 CE sequence derived features may provide similar results^2^.

Feature reduction is an integral part of model building process. An important finding of our study was the higher performance of embedded classifier models (LASSO and elastic net) compared to a priori feature reduction. Embedded methods perform feature extraction at the time of model training process. Embedded models have advantage in terms of better generalizability than filter and wrapper methods. They are also quick and computationally less extensive compared to wrapper methods^34^. A priori filter feature reduction methods may lead to loss of relevant features with a drop in model performance as seen in our study with high correlation and linear combination filter reduction methods. Although, few of the top models also included filter-based methods (Tables 2, 3), most of the top performing models were models with embedded feature selection.

In relation to the mask performance, we found that the most important features were often extracted both from the whole tumor and edema masks. This was true for all sequence combinations (mp-MRI, individual sequence, and from combined T1-CE and FLAIR sequence). Additionally, the most important radiomics feature was shape sphericity. This is likely secondary to different peritumoral environment in GBM and IMD. Since GBM is irregular with variable growth in different dimensions while IMD often has well-defined margins and a more uniform shape, it is not surprising that shape sphericity was the most important discriminating feature. Additionally, the peritumoral region in patients with IMD is invariably vasogenic edema while GBM shows neoplastic infiltration^2^. This may explain the high performance of edema masks. Few prior studies have also shown morphometrical analysis and peritumoral radiomic features as important parameters to differentiate GBM and IMD^35,36^.

It is pertinent to note here that the model performance did not change when using FLAIR only, as compared to FLAIR and T1-CE in our study. Also, the performance of FLAIR and combined FLAIR and T1-CE sequence was comparable to mp-MRI. Even though it may appear that FLAIR sequence alone might suffice in terms of providing robust discrimination, it is pertinent to note here that the lesion segmentation between enhancing tumor and surrounding ‘edema’ requires both T1 CE and FLAIR images to generate the masks. At the very minimum therefore, both these sequences would be required for differentiating between GBM and IMD and achieving performance comparable to mp-MRI. This may also be relevant for future validation studies as analysis of one or few MRI sequences is quicker and is easier to implement in terms of time and computational costs. Prior radiomics studies have only assessed single or few MRI sequences for GBM and metastasis classification. Our study provides evidence of comparable diagnostic performance of single MRI sequence versus mp-MRI.

There were several limitations in our study. This was a single center study with limited sample size, and we did not perform external validation of our best performing model that could further establish model generalizability. Secondly, deep neural networks were not evaluated in our study. Another limitation was a less heterogenous group of brain metastases, since we only included two of the most common primary sites of malignancies (lung and breast). It is possible that increasing radiomic heterogeneity could negatively impact model performance. However, since shape sphericity was the most useful feature and is likely to remain consistent across metastasis as a tumor-class, the impact of additional sub-types of metastasis may not be significant.

However, our study had several strengths including radiomics assessment from both mp-MRI and individual or sequence combinations, different tumor and edema masks and comparison of forty-five different classifier model and feature reduction combinations for each sequence/ combination of sequences. Our study also has one of the best model performances reported for this specific two-class problem. Finally, our results would favor using embedded feature selection over a priori feature reduction.

## Conclusion

Radiomics based machine learning can classify GBM and IMD with excellent diagnostic performance. Model performance can vary depending upon the chosen classifier and feature reduction combination and thus comprehensive model selection strategy should be chosen. The performance of mp-MRI and single FLAIR or combined T1-CE and FLAIR sequence is comparable. Radiomic features with higher relative importance came from both whole tumor and edema masks with shape sphericity being the most important feature across multiple models.

## Materials and methods

This was a retrospective study approved by institutional review board (IRB) of University of Iowa Hospitals and requirement of informed consent was waived off by University of Iowa Hospitals’ IRB (IRB-ID 201912239). The study was carried out in accordance with relevant guidelines and regulations. Between 2010 and 2016, patients were identified from institutional cancer registries and electronic medical records. Eligibility criteria included availability of pre-therapeutic/ pre-operative multiparametric MRI (mp-MRI) images (T1W, T2W, FLAIR, ADC, T1-contrast enhanced (T1-CE), and the presence of a contrast-enhancing tumor measuring greater than 1 cm in at least one axial dimension. Patients with non-enhancing tumors and motion artifact were excluded. This yielded a total of 60 patients with GBM. A similar number of patients (n = 60) with known lung (n = 40) or breast (n = 20) primary tumor and intracranial metastatic disease were then consecutively selected from the same time period, again using a combination of institutional registries and electronic medical records. In patients with multiple lesions, only the largest lesion was segmented since this approach can provide reliable results by including regions containing a sufficient number of voxels and the same approach has also been utilized in prior studies^26,37^.

### Image acquisition

Preoperative imaging was performed on 1.5 T MRI system (Siemens, Erlangen, Germany). The acquisition protocol for brain tumor evaluation at our hospital includes pre-contrast axial T1W, T2W, FLAIR, DWI with ADC maps, gradient echo and tri-planar T1-CE images (details in supplementary Table S5).

### Image pre-processing

DICOM images were first anonymized and converted to NIfTI format. All images were resampled to 1 × 1 × 5 mm^3^ voxel size using the AFNI package (https://afni.nimh.nih.gov/)^38^. All image sequences acquired during same session were mutually registered to the T1W sequence using Advanced Normalization Tools (ANTs) (http://stnava.github.io/ANTs/)^39^. After image resampling and registration, intensity normalization was performed to [0,255] using the feature scaling method available in the ANTs registration suite.

### Tumor segmentation

3-D tumor segmentation was performed on axial T1-CE and FLAIR images by two radiologists (SP, GB) in consensus using an in-house developed semi-automatic tool ‘Layered Optimal Graph Image Segmentation for Multiple Objects and Surfaces’ (LOGISMOS)^40^ that first automatically identifies the tumor surfaces followed by an efficient ‘just-enough interaction’ with an optional surface editing step which may be invoked if needed. Two separate region of interests (masks) were created using T1-CE and FLAIR images: i) whole tumor (enhancing plus necrotic); and ii) peritumoral edema. First, whole tumor mask was generated from the T1-CE sequence. This was followed by generation of a mask from FLAIR images that included the entire lesion (whole tumor and surrounding edema). Finally, edema mask was extracted by subtracting the T1-CE derived whole tumor mask from FLAIR derived entire lesion mask. Both whole tumor and edema masks were expert-identified. These masks were then superimposed on all five MRI sequences.

### Texture features extraction

Features were extracted using Pyradiomics 3.0^41^. For each tumor, features were abstracted using above two masks and on each mask five MRI sequences were used. This resulted in 10 possible masks and sequence combinations, on each of which 107 radiomic features were obtained, yielding a total of 1070 features.

Each set of 107 features included: 3D shape features (n = 14), first order features (n = 18), gray level co-occurrence matrix features (n = 24), gray level dependency matrix features (n = 14), gray level run length matrix features (n = 16), gray level size zone matrix features (n = 16) and neighboring gray tone difference matrix features (n = 5). The default value for the number of bins was fixed by bin width of 25 Gy levels. In rare cases where the edema was minimal (7 patients (6%)) leading to absence of a corresponding mask, the value of the corresponding feature was set to -9999.

### Feature reduction

Since the number of features exceeded the sample size, feature reduction was performed to reduce noise, collinearity and dimensionality. Three feature reduction methods were considered: a linear combinations filter, a high correlation filter, and principal components analysis (PCA). The linear combinations (lincomb) filter addresses both collinearity and dimension reduction by finding linear combinations of two or more variables and removing columns to resolve the issue. This process is repeated until the feature set is full rank. The high correlation (corr) filter removes variables from the feature set which have a large absolute correlation. A user-specified threshold is given to determine the largest allowable absolute correlation (threshold 0.4 for mp-MRI, 0.6 for individual sequence, 0.5 for combined T1-CE and FLAIR). Lower thresholds were used as the number of potential features increased for consistency in the number of potential features after filtering. These thresholds led to 60–80 features remaining after high correlation filtering across all potential feature sets.

The number of components retained in the PCA transformation was determined by specifying the fraction of the total variance that should be covered by the components (80% threshold for mp-MRI and 85% for both individual sequence and combined T1-CE and FLAIR). These thresholds were chosen to sufficiently reduce the dimensionality of the feature set for model fitting while retaining many of the important variables and as with the high correlation filter, different thresholds were used to ensure similar numbers of PC’s being used in the modeling process. These thresholds led to 25–30 PC’s used to be used in model fitting. These feature reduction methods were implemented using the recipes package in R version 4.0.2^42,43^. All variables were standardized, and missing data was imputed using mean imputation prior to any feature reduction. Mean imputation was used for simplicity as the amount of missing data was small. The feature reduction techniques, feature standardization, and imputation were carried out within each cross-validated split of the data, so as not to bias the estimate of predictive performance.

### Model fitting

Twelve different machine learning models were fit to determine the best classifier for each feature set. These models can be broadly classified as: linear classifiers, non-linear classifiers, and ensemble classifiers. The linear classifiers used were linear, logistic, ridge, elastic net (enet), and LASSO (least absolute shrinkage and selection operator) regression. The non-linear classifiers used were neural network, support vector machine with a polynomial kernel (svmPoly), SVM with a radial kernel (svmRad), and multi-layer perceptron (MLP). Finally, the ensemble classifiers used were random forest, generalized boosted regression model (GBRM), and boosting of classification trees with adaBoost.

### Classifier model performance evaluation

Each model was fit using the three feature reduction techniques as well as the entire feature set (full), except for the linear regression, logistic regression, and the neural network which cannot be fit with the full feature set due to numerical stability (linear and logistic) and computational complexity (neural network). The entire feature set was used to determine if embedded feature selection of the machine learning model itself performed better than a priori feature reduction using either of the three feature reduction techniques. This yielded 45 possible model/feature reduction combinations to be fit to each of the possible feature sets. A fivefold repeated cross-validation with five repeats was then performed to evaluate the predictive performance of each model, giving 25 total estimates of prediction performance for each model/feature reduction combination.

For models with tuning parameters, the important parameters were tuned using nested cross-validation, which has been shown to provide unbiased performance estimates regardless of the sample size^46^. Tuning of important parameters was handled using the functionality of the MachineShop R package^44^. Tuning grids across 5 different values for each tuning parameter were used, except for the MLP model which used a grid across 3 different values for each tuning parameter due to the computational complexities of that model. Other parameters were set to the package default values, which can be found in the online documentation for each model constructor function^45^. For example, for the random forest model, the number of trees was 500 (default), the number of variables randomly sampled as candidates at each split was tuned across a grid of five possible values, and the minimum size of the terminal nodes was 1 (default). Although the MachineShop package offers users the ability to choose the values in the tuning grid, we utilized the functionality of the package which automatically generates the grid of possible values for the tuning parameters due to the large number of models under consideration.

Nested Cross-validation was implemented using the “resample” function in the MachineShop package using the “TunedModel” constructor function to perform model tuning within each fold. This function automatically creates cross-validated splits using different seeds, and an overall seed is provided to ensure the splits are the same for each candidate model. Table 5 provides the MachineShop model constructor function name, the parameters that were tuned, and the total length of the tuning grid for each of the models we considered. The MLP is not implemented by default in MachineShop but was implemented using the package’s ability for users to define a custom model to ensure it was evaluated on the same cross-validated splits as all of the other models.

**Table 5:** Summary of tuning for each model.

Model	MachineShop Model constructor	Parameters tuned	Size of tuning grid
Linear	LMModel	N/A	–
Logistic	GLMModel	N/A	–
Ridge	GLMNetModel Alpha = 0	Lambda	5
Elastic Net	GLMNetModel	Alpha, Lambda	25
LASSO	GLMNetModel Alpha = 1	Lambda	5
Neural Network	NNetModel	Size, Decay	25
SVM Poly	SVMPolyModel	C, Degree, Scale	125
SVM Radial	SVMRadialModel	C, Sigma	25
MLP	N/A	Size, Maxit, learnFuncParams	27
Random Forest	RandomForestModel	Mtry	5
GBRM	GBMModel	n.trees, interaction.depth	25

LASSO: (least absolute shrinkage and selection operator); SVM Poly: support vector machine with a polynomial kernel; SVM Radial; MLP: multi-layer perceptron; LMModel: linear model; GLMModel: generalized linear model; GLMNetModel: generalized linear model with penalized maximum likelihood; NNetModel: feed-forward neural networks; GBMModel: generalized boosted regression; Lambda: regularization parameter; Alpha: elastic net mixing parameter; Size: number of units in the hidden layer(s); Decay: parameter for weight decay; C: cost of constraints violation, regularization term in the Lagrange formulation; Degree: degree of the polynomial kernel function; Scale: scaling parameter of the polynomial kernel; Sigma: inverse kernel width; Maxit: maximum iterations to learn; learnFuncParams: parameters of the learning function; Mtry: number of variables randomly sampled as candidates at each split; n.trees: total number of trees to fit; interaction.depth: maximum depth of variable interactions.

### Feature importance

Feature importance was calculated for the top models using the final model fit to the entire data set from each of the three scenarios. Feature importance for the LASSO and elastic net models was defined as the absolute value of the regression coefficients. As the features are standardized, larger values of the coefficients correspond to higher importance. For the adaBoost model, the feature importance was determined by finding the improvement in the Gini index attributed to a split on that feature in a tree and the weight of that tree^47^. Importance values were rescaled to range from 0 to 100, with the most important feature having a value of 100.

### Statistical analysis

The goal of this analysis was to determine the best model for tumor classification and to determine if models fit using all five MRI sequences (mp-MRI) performed better than models fit to the individual sequences or models fit using combined T1-CE and FLAIR sequences. For the models using mp-MRI, there were 1070 possible features (2 masks × 5 sequences × 107 features). For each sequence specific model, the feature set included 214 (2 masks × 1 sequence × 107 features) possible features, and for the T1-CE and FLAIR combination models, the feature set included 428 (2 masks × 2 sequences × 107 features) possible features.

Model fitting and cross-validated predictive performance was implemented using the MachineShop and RSNNS packages in R version 4.0.2^44^. Predictive performance was measured with the area under the receiver operating characteristic curve (AUC) for interpretability. As models were formulated to predict GBM, AUC estimates the probability that a randomly selected subject that had a GBM will have a greater predicted value than a randomly selected subject that had a metastatic tumor. Higher AUC values indicate better predictive performance. The mean AUC between each of these three models was compared using a paired t-test. We provide the mean/SD of the AUC over the 25 cross-validated estimates to describe the distribution of AUC estimates.

## Supplementary Information

Supplementary Information.

## Data Availability

The datasets generated during and/or analyzed during the current study are available from the corresponding author on reasonable request.
