# Supplementary material for: Machine learning based differentiation of glioblastoma from brain metastasis using MRI derived radiomics
Source: Sci Rep. 2021 May 18;11:10478. doi: 10.1038/s41598-021-90032-w (PMC8131619; doi:10.1038/s41598-021-90032-w)
Supplement: Supplementary file 1 — Supplementary Information. [file 41598_2021_90032_MOESM1_ESM.pdf]

TITLE Page

Machine Learning based Differentiation of Glioblastoma from Brain Metastasis using MRI derived Radiomics

Author names and affiliations

Sarv Priya[1], MBBS

Yanan Liu[2], PhD

Caitlin Ward[3], MS

Nam H Le[2] MS

Neetu Soni[1] MD

Ravishankar Pillenahalli Maheshwarappa[1] MD

Varun Monga[4], MD

Honghai Zhang[2], PhD

Milan Sonka[2], PhD

Girish Bathla[1] MBBS, FRCR

1: Department of Radiology, University of Iowa Hospitals and Clinics, Iowa City, IA, USA

2: College of Engineering, University of Iowa, Iowa City, IA, USA

3: Department of Biostatistics, University of Iowa, Iowa City, IA, USA

4: Department of Medicine, University of Iowa Hospitals and Clinics, Iowa City, IA, USA

Type of manuscript: Original research

Correspondence:

Sarv Priya MD

Department of Radiology,

University of Iowa Hospital and Clinics,

200 Hawkins Drive

Iowa City, IA, 52242

E-mail: sarv-priya@uiowa.edu

Phone: 319-512-5404

Supplementary Table S1: Ranking by variable Importance for the 10 most important features in the two best models fit using all sequences

	LASSO Full Feature set			
**Rank**	**Mask**	**Seq**	**Feature**	**Relative Importance**
1	WT	F	shape_Sphericity	100.00
2	WT	T2	Maximum2DDiameterRow	49.63
3	WT	CE	firstorder_Skewness	39.31
4	WT	T1	gldm_SmallDependenceHighGrayLevelEmphasis	34.16
5	Edema	A	ngtdm_Contrast	31.87
6	Edema	F	shape_Sphericity	26.28
7	Edema	T1	firstorder_10Percentile	17.36
8	WT	T1	shape_LeastAxisLength	17.04
9	WT	F	firstorder_RobustMeanAbsoluteDeviation	16.14
10	Edema	T2	shape_LeastAxisLength	12.70
	**Elastic Net Full Feature set**			
**Rank**	**Mask**	**Seq**	**Feature**	**Relative Importance**
1	WT	F	shape_Sphericity	100.00
2	WT	CE	firstorder_Skewness	24.24
3	Edema	A	ngtdm_Contrast	24.02
4	Edema	F	shape_Sphericity	21.67
5	WT	T2	firstorder_InterquartileRange	19.66
6	Edema	A	Maximum2DDiameterRow	18.36
7	WT	T1	gldm_SmallDependenceHighGrayLevelEmphasis	18.16
8	WT	T2	glcm_Correlation	17.79
9	WT	F	firstorder_RobustMeanAbsoluteDeviation	17.35
10	Edema	T1	firstorder_Kurtosis	13.28

WT: Whole tumor mask; A: ADC; F: FLAIR; CE: T1-CE

Supplementary Figure 1: Feature ranking for the 10 most important features in the two best models fit using all sequences

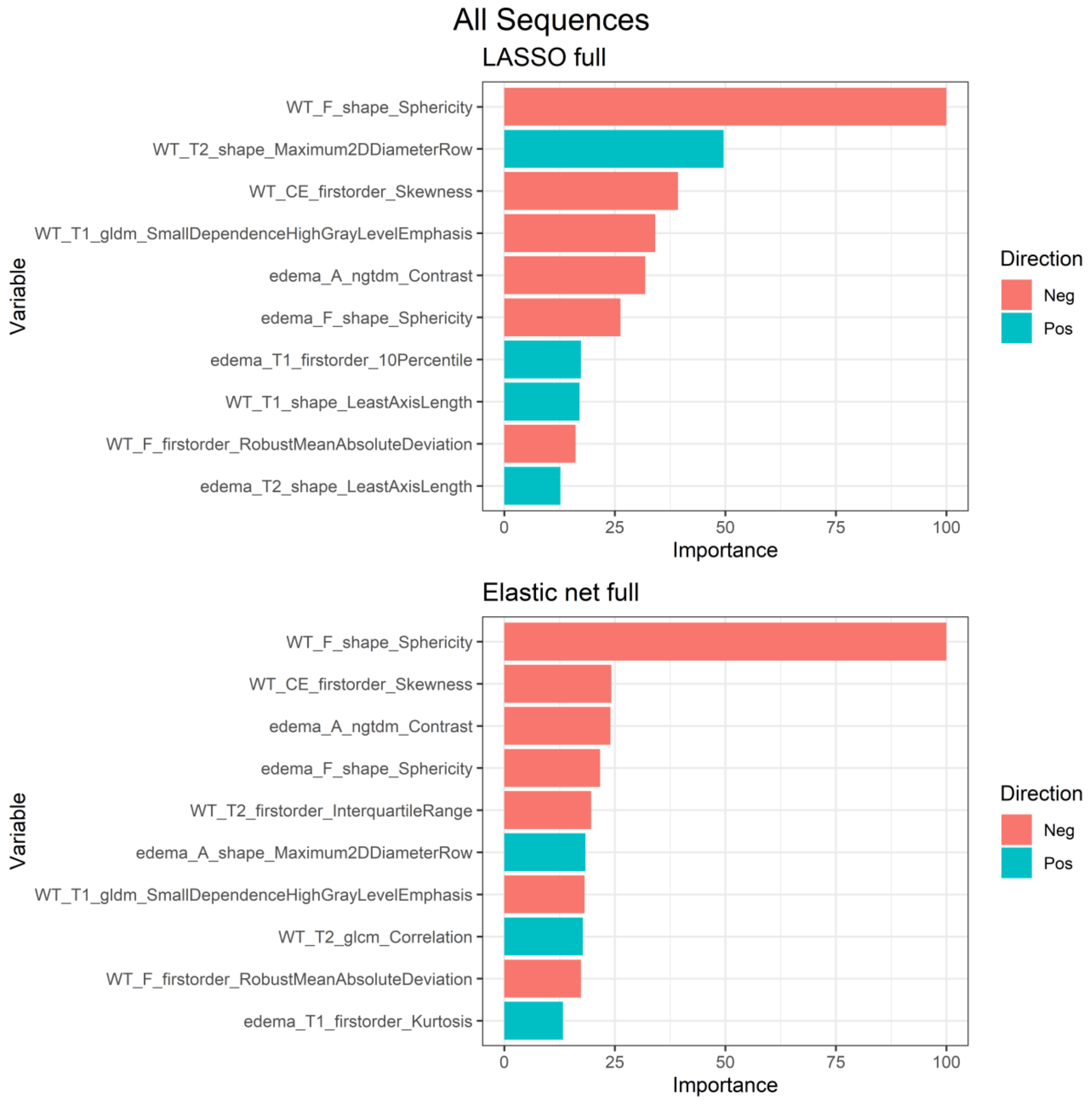

Positive (Pos) association indicates that larger value of that feature is associated with GBM and a negative (Neg) value indicates that smaller values of that feature are associated with GBM (i.e. larger values are associated with metastasis).

Supplementary Table S2: Ranking by variable Importance for the 10 most important features in the two best models fit using individual sequences

	**LASSO Full Feature set FLAIR**		
**Rank**	**Mask**	**Feature**	**Relative Importance**
1	WT	shape_Sphericity	100.00
2	Edema	Maximum2DDiameterRow	38.46
3	Edema	firstorder_InterquartileRange	33.00
4	Edema	glszm_SmallAreaHighGrayLevelEmphasis	31.07
5	WT	glszm_SmallAreaHighGrayLevelEmphasis	26.64
6	Edema	firstorder_RobustMeanAbsoluteDeviation	21.92
7	WT	firstorder_RobustMeanAbsoluteDeviation	19.74
8	Edema	gldm_LargeDependenceLowGrayLevelEmphasis	16.80
9	WT	firstorder_TotalEnergy	14.84
10	WT	glrlm_ShortRunHighGrayLevelEmphasis	11.75
	**Elastic Net Full Feature set FLAIR**		
**Rank**	**Mask**	**Feature**	**Relative Importance**
1	WT	shape_Sphericity	100.00
2	Edema	Maximum2DDiameterRow	38.46
3	Edema	firstorder_InterquartileRange	33.00
4	Edema	glszm_SmallAreaHighGrayLevelEmphasis	31.07
5	WT	glszm_SmallAreaHighGrayLevelEmphasis	26.64
6	Edema	firstorder_RobustMeanAbsoluteDeviation	21.92
7	WT	firstorder_RobustMeanAbsoluteDeviation	19.74
8	Edema	gldm_LargeDependenceLowGrayLevelEmphasis	16.80
9	WT	firstorder_TotalEnergy	14.84
10	WT	glrlm_ShortRunHighGrayLevelEmphasis	11.75

WT: Whole tumor mask

Supplementary Figure 2: Feature ranking for the 10 most important features in the two best models fit using individual sequences

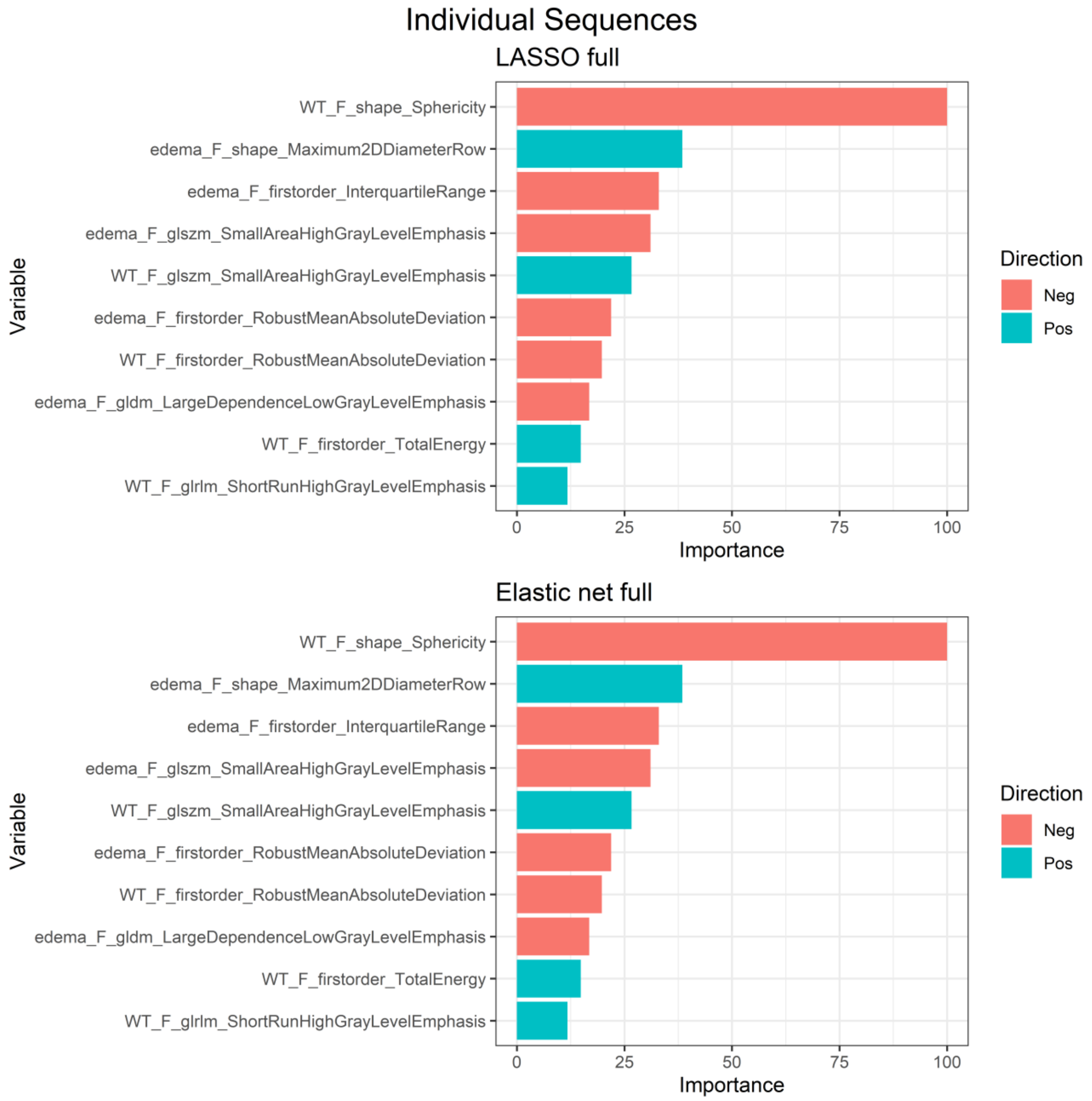

Positive (Pos) association indicates that larger value of that feature is associated with GBM and a negative (Neg) value indicates that smaller values of that feature are associated with GBM (i.e. larger values are associated with metastasis).

Supplementary Table S3: Ranking by variable Importance for the 10 most important features in the two best models fit using T1-CE and FLAIR sequences

	**adaBoost Full T1 CE+F**			
**Rank**	**Mask**	**Seq**	**Feature**	**Relative Importance**
1	WT	F	shape_Sphericity	100.00
2	Edema	F	gldm_LargeDependenceHighGray LevelEmphasis	92.81
3	WT	F	glrlm_LongRunEmphasis	43.11
4	WT	F	firstorder_TotalEnergy	39.19
5	WT	CE	glcm_Imc1	39.11
6	WT	F	shape_MajorAxisLength	31.76
7	WT	CE	glszm_SmallAreaEmphasis	31.03
8	Edema	CE	glcm_Contrast	28.13
9	Edema	CE	shape_Maximum2DDiameterRow	25.58
10	WT	F	gldm_SmallDependenceEmphasis	25.44
	**LASSO Full T1 CE+F**			
**Rank**	**Mask**	**Seq**	**Feature**	**Relative Importance**
1	WT	F	shape_Sphericity	100.00
2	Edema	CE	shape_Maximum2DDiameterRow	40.61
3	Edema	F	glszm_SmallAreaHighGrayLevelEmphasis	34.69
4	Edema	F	firstorder_InterquartileRange	30.52
5	WT	CE	firstorder_Skewness	30.31
6	Edema	F	shape_Sphericity	20.48
7	WT	CE	glszm_SmallAreaEmphasis	20.34
8	WT	F	firstorder_RobustMeanAbsoluteDeviation	19.99
9	WT	CE	ngtdm_Busyness	17.83
10	Edema	F	firstorder_RobustMeanAbsoluteDeviation	12.91

WT: Whole tumor mask; A: ADC; F: FLAIR; CE: T1-CE

Supplementary Figure 3: Feature ranking for the 10 most important features in the two best models fit using T1-CE and FLAIR sequences

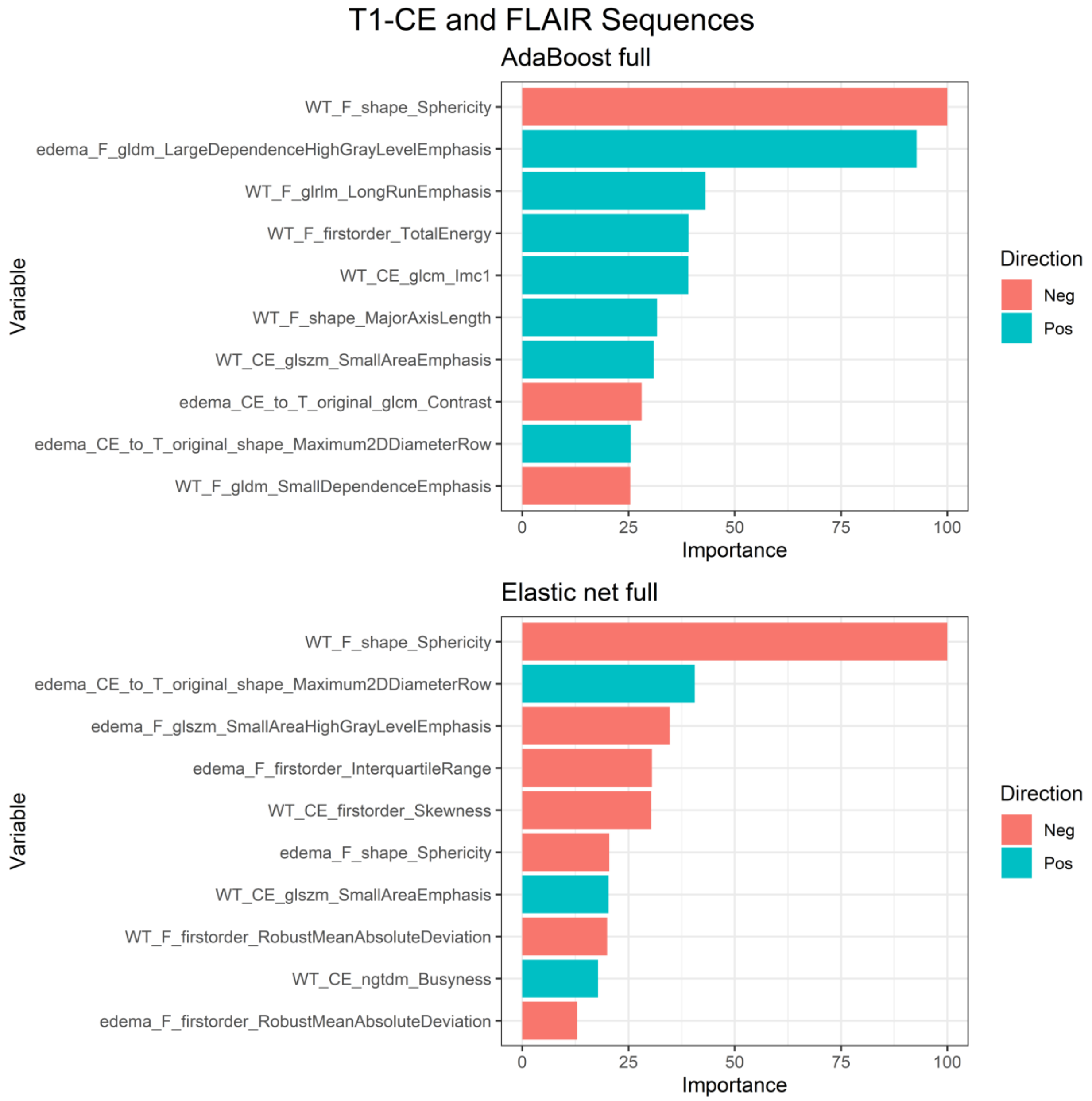

Positive (Pos) association indicates that larger value of that feature is associated with GBM and a negative (Neg) value indicates that smaller values of that feature are associated with GBM (i.e. larger values are associated with metastasis).

Supplementary Figure 4: Heatmap for feature importance for multiparametric MRI models

Red (positive) is indicative that larger values are associated with Glioblastoma and the blue (negative) is indicative that smaller values are associated with Glioblastoma (i.e. larger values are associated with metastasis).

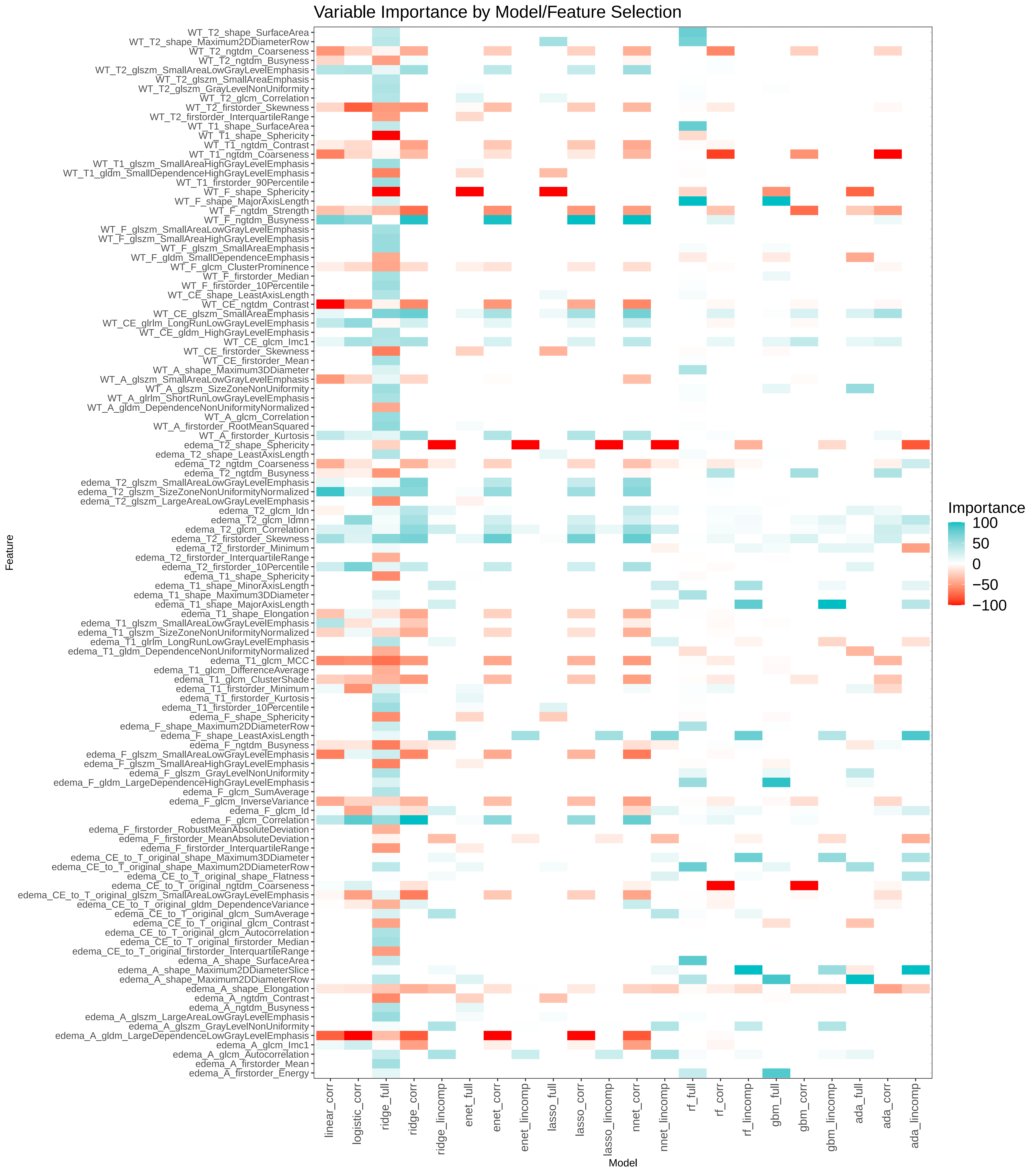
Variable Importance by Model/Feature Selection
WT_T2_shape_SurfaceArea
WT_T2_shape_Maximum2DDiameterRow
WT_T2_ngtdm_Coarseness
WT_T2_ngtdm_Busyness
WT_T2_glszm_SmallAreaLowGrayLevelEmphasis
WT_T2_glszm_SmallAreaEmphasis
WT_T2_glszm_GrayLevelNonUniformity
WT_T2_glcm_Correlation
WT_T2_firstorder_Skewness
WT_T2_firstorder_InterquartileRange
WT_T1_shape_SurfaceArea
WT_T1_shape_Sphericity
WT_T1_ngtdm_Contrast
WT_T1_ngtdm_Coarseness
WT_T1_glszm_SmallAreaHighGrayLevelEmphasis
WT_T1_gldm_SmallDependenceHighGrayLevelEmphasis
WT_T1_firstorder_90Percentile
WT_F_shape_Sphericity
WT_F_shape_MajorAxisLength
WT_F_ngtdm_Strength
WT_F_ngtdm_Busyness
WT_F_glszm_SmallAreaLowGrayLevelEmphasis
WT_F_glszm_SmallAreaHighGrayLevelEmphasis
WT_F_glszm_SmallAreaEmphasis
WT_F_gldm_SmallDependenceEmphasis
WT_F_glcm_ClusterProminence
WT_F_firstorder_Median
WT_F_firstorder_10Percentile
WT_CE_shape_LeastAxisLength
WT_CE_ngtdm_Contrast
WT_CE_glszm_SmallAreaEmphasis
WT_CE_glrlm_LongRunLowGrayLevelEmphasis
WT_CE_gldm_HighGrayLevelEmphasis
WT_CE_glcm_Imc1
WT_CE_firstorder_Skewness
WT_CE_firstorder_Mean
WT_A_shape_Maximum3DDiameter
WT_A_glszm_SmallAreaLowGrayLevelEmphasis
WT_A_glszm_SizeZoneNonUniformity
WT_A_glrlm_ShortRunLowGrayLevelEmphasis
WT_A_gldm_DependenceNonUniformityNormalized
WT_A_glcm_Correlation
WT_A_firstorder_RootMeanSquared
WT_A_firstorder_Kurtosis
edema_T2_shape_Sphericity
edema_T2_shape_LeastAxisLength
edema_T2_ngtdm_Coarseness
edema_T2_ngtdm_Busyness
edema_T2_glszm_SmallAreaLowGrayLevelEmphasis
edema_T2_glszm_SizeZoneNonUniformityNormalized
edema_T2_glszm_LargeAreaLowGrayLevelEmphasis
edema_T2_glcm_Idn
edema_T2_glcm_Idmn
edema_T2_glcm_Correlation
edema_T2_firstorder_Skewness
edema_T2_firstorder_Minimum
edema_T2_firstorder_InterquartileRange
edema_T2_firstorder_10Percentile
edema_T1_shape_Sphericity
edema_T1_shape_MinorAxisLength
edema_T1_shape_Maximum3DDiameter
edema_T1_shape_MajorAxisLength
edema_T1_shape_Elongation
edema_T1_glszm_SmallAreaLowGrayLevelEmphasis
edema_T1_glszm_SizeZoneNonUniformityNormalized
edema_T1_glrlm_LongRunLowGrayLevelEmphasis
edema_T1_gldm_DependenceNonUniformityNormalized
edema_T1_glcm_MCC
edema_T1_glcm_DifferenceAverage
edema_T1_glcm_ClusterShade
edema_T1_firstorder_Minimum
edema_T1_firstorder_Kurtosis
edema_T1_firstorder_10Percentile
edema_F_shape_Sphericity
edema_F_shape_Maximum2DDiameterRow
edema_F_shape_LeastAxisLength
edema_F_ngtdm_Busyness
edema_F_glszm_SmallAreaLowGrayLevelEmphasis
edema_F_glszm_SmallAreaHighGrayLevelEmphasis
edema_F_glszm_GrayLevelNonUniformity
edema_F_gldm_LargeDependenceHighGrayLevelEmphasis
edema_F_glcm_SumAverage
edema_F_glcm_InverseVariance
edema_F_glcm_Id
edema_F_glcm_Correlation
edema_F_firstorder_RobustMeanAbsoluteDeviation
edema_F_firstorder_MeanAbsoluteDeviation
edema_F_firstorder_InterquartileRange
edema_CE_to_T_original_shape_Maximum3DDiameter
edema_CE_to_T_original_shape_Maximum2DDiameterRow
edema_CE_to_T_original_shape_Flatness
edema_CE_to_T_original_ngtdm_Coarseness
edema_CE_to_T_original_glszm_SmallAreaLowGrayLevelEmphasis
edema_CE_to_T_original_gldm_DependenceVariance
edema_CE_to_T_original_glcm_SumAverage
edema_CE_to_T_original_glcm_Contrast
edema_CE_to_T_original_glcm_Autocorrelation
edema_CE_to_T_original_firstorder_Median
edema_CE_to_T_original_firstorder_InterquartileRange
edema_A_shape_SurfaceArea
edema_A_shape_Maximum2DDiameterSlice
edema_A_shape_Maximum2DDiameterRow
edema_A_shape_Elongation
edema_A_ngtdm_Contrast
edema_A_ngtdm_Busyness
edema_A_glszm_LargeAreaLowGrayLevelEmphasis
edema_A_glszm_GrayLevelNonUniformity
edema_A_gldm_LargeDependenceLowGrayLevelEmphasis
edema_A_glcm_Imc1
edema_A_glcm_Autocorrelation
edema_A_firstorder_Mean
edema_A_firstorder_Energy
Feature
linear_corr
logistic_corr
ridge_full
ridge_corr
ridge_lincomp
enet_full
enet_corr
enet_lincomp
lasso_full
lasso_corr
lasso_lincomp
nnet_corr
nnet_lincomp
rf_full
rf_corr
rf_lincomp
gbm_full
gbm_corr
gbm_lincomp
ada_full
ada_corr
ada_lincomp
Model
Importance
100
50
0
−50
−100

Supplementary Table S4: Comparison with prior radiomic studies differentiating glioblastoma from metastatic brain lesions

Authors	Patients	Scanner/sequences/Masks	Texture	ML/DL	Accuracy
**Priya et al (Current study)**	120 (GBM-60, Metastasis 60)	1.5 T; volumetric 3D segmentation; whole tumor and edema mask;	107 radiomics features	12 machine learning models and three feature selection methods	LASSO (using full feature set); AUC 0.953
Dong et al 2020[1]	120 (GBM-60, Metastasis 60)	1.5t/3t; 2-D; T1 CE, T2W, T1W; edema mask	321 features	Boruta algorithm, 5 classifiers	Highest accuracy (77%)-achieved after combining all 5 ML classifiers
Ortiz et al 2020[2]	100 (GBM-50, Metastasis 50)	1.5T; 2-D; T1 CE coronal; only largest tumor segmented; enhancing mask	88 rotation invariant features	5 classifier models, 3 feature selection methods	MLP model- Accuracy (82%); AUC- 0.912
Bae et al 2020[3]	248 (GBM-159, Metastasis 89)	3-T; 2-D; T1 CE; T2W; enhancing, edema masks, enhancing plus edema combined	265	5 methods for feature selection; 7 machine learning classifiers; Deep learning- Deep neural network (DNN)	DNN- Highest accuracy from combined mask (89%); AUC (0.956);<br><br>Best ML model – from combined masks (adaptive boosting combined with tree-based feature selection- AUC- 0.890; Accuracy 83%
Csutak et al 2020[4]	42 (High grade glioma-16, Metastasis 26)	1.5 T, T2W, peritumoral texture	First, second order, wavelet texture features	Fisher selection method	Perc01 texture feature yielded highest AUC (0.858)

Tateishi et al 2020[5]	126 (GBM-73, Metastasis 53)	3T, T1CE, T2W, ADC	36 Features	SVM	AUC 0.920
Dastmalchian et al 2020[6]	31 (GBM-17, Low grade glioma-6, Metastasis-8	3T, T1 and T2 maps, MRI fingerprinting	39 Texture features	Log rank test	AUC 0.877 (GBM versus metastasis)
Artzi et al 2019[7]	439 (GBM-212, IMD- 227)	1.5T/3T; 2-D; T1 CE; Three tumor slices selected; enhancing mask	First, Second-order, morphological, wavelet features	Supervised learning-4 ML classifiers-Unsupervised learning-Bag of features (BoF) extraction	Supervised-SVM- 85% (AUC-0.940) Unsupervised - BoF Accuracy-75%
Qian et al 2019[8]	412 (GBM 242, Metastasis 170)	3T; 2-D; T1 CE; volumetric segmentation	1303 radiomic features	12 feature selection methods; 7 supervised machine-learning classifiers	LASSO-SVM-Accuracy-83% (AUC 0.900)
Zhang et al 2019[9]	62 (GBM-36, Metastasis 26)	3T; 2-D; ADC; largest cross-section only; whole tumor mask (enhancing plus necrotic); enhancing mask only	5 GLCM parameters	No	Whole tumor mask- AUC 0.682 (Homogeneity texture); enhancing only mask (AUC 0.886; Homogeneity texture)
Skogen et al 2019[10]	43 (GBM-22, Metastasis 21)	3T; 2-D; ADC, FA maps; largest cross-section only segmented;	5 first-order texture features at different filters	No	FA plus ADC unfiltered image- AUC-0.911
Petrujkić et al 2019[11]	55 (GBM-30, Metastasis 25)	3T, R2W, Susceptibility weighted imaging, T1 CE	Fractal and texture analysis	No	AUC 0.908
Mouthuy et al 2012[12]	50 (GBM-41, Metastasis 14)	1.5 T, peritumoral texture from washout phase of the perfusion sequence	9 texture features	k-means clustering	Highest AUC 0.75 from correlation texture

					feature

ML- machine learning; DL: deep learning; LASSO: least absolute shrinkage and selection operator; SVM: support vector machine; MLP: multilayer perceptron; FA: fractional anisotropy

Supplementary Table S5: Typical scanning parameters for various sequences at the authors institute:

T1W (TR/TE/TI: 1950/10/840, NEX: 2, slice thickness: 5 mm, matrix: 320 × 256, field of view (FOV) 240 mm, pixel size 0.75 mm)

T2W (TR/TE: 4000/90, NEX: 2, slice thickness: 5 mm, matrix: 512 × 408, FOV 240 mm, pixel size 0.5 mm);

FLAIR (TR/TE/TI: 9000/105/2500, NEX: 1, slice thickness: 5 mm, matrix: 384 × 308, FOV 240 mm, pixel size 0.6 mm)

DWI (TR/TE: 4000/74, NEX: 3, slice thickness: 5 mm, matrix: 128 × 128, FOV 240 mm, pixel size 1.8 mm)

T1W-CE (TR/TE/TI: 570/13, NEX: 2, slice thickness: 5 mm, matrix: 384 × 312, field of view (FOV) 240 mm, pixel size 0.62 mm)

T1 W contrast-enhanced images were acquired after administration of gadobenate dimeglumine (Multihance; Bayer Healthcare Pharma) or gadobutrol (Gadavist; Bayer Healthcare Pharma, Berlin, Germany) injected at the rate of 0.1 ml/kg body weight.

References:

1 Dong, F. *et al.* Differentiation of supratentorial single brain metastasis and glioblastoma by using peri-enhancing oedema region-derived radiomic features and multiple classifiers. *European radiology* **30**, 3015-3022, doi:10.1007/s00330-019-06460-w (2020).

2 Ortiz-Ramón, R., Ruiz-España, S., Mollá-Olmos, E. & Moratal, D. Glioblastomas and brain metastases differentiation following an MRI texture analysis-based radiomics approach. *Physica medica : PM : an international journal devoted to the applications of physics to medicine and biology : official journal of the Italian Association of Biomedical Physics (AIFB)* **76**, 44-54, doi:10.1016/j.ejmp.2020.06.016 (2020).

3 Bae, S. *et al.* Robust performance of deep learning for distinguishing glioblastoma from single brain metastasis using radiomic features: model development and validation. *Scientific reports* **10**, 12110, doi:10.1038/s41598-020-68980-6 (2020).

4 Csutak, C. *et al.* Differentiating High-Grade Gliomas from Brain Metastases at Magnetic Resonance: The Role of Texture Analysis of the Peritumoral Zone. *Brain sciences* **10**, doi:10.3390/brainsci10090638 (2020).

5 Tateishi, M. *et al.* An initial experience of machine learning based on multi-sequence texture parameters in magnetic resonance imaging to differentiate glioblastoma from brain metastases. *Journal of the neurological sciences* **410**, 116514, doi:10.1016/j.jns.2019.116514 (2020).

6 Dastmalchian, S. *et al.* Radiomic analysis of magnetic resonance fingerprinting in adult brain tumors. *European journal of nuclear medicine and molecular imaging*, doi:10.1007/s00259-020-05037-w (2020).

7 Artzi, M., Bressler, I. & Ben Bashat, D. Differentiation between glioblastoma, brain metastasis and subtypes using radiomics analysis. *Journal of magnetic resonance imaging : JMRI* **50**, 519-528, doi:10.1002/jmri.26643 (2019).

8 Qian, Z. *et al.* Differentiation of glioblastoma from solitary brain metastases using radiomic machine-learning classifiers. *Cancer letters* **451**, 128-135, doi:10.1016/j.canlet.2019.02.054 (2019).

9 Zhang, G. *et al.* Discrimination Between Solitary Brain Metastasis and Glioblastoma Multiforme by Using ADC-Based Texture Analysis: A Comparison of Two Different ROI Placements. *Academic radiology* **26**, 1466-1472, doi:10.1016/j.acra.2019.01.010 (2019).

10 Skogen, K. *et al.* Texture analysis on diffusion tensor imaging: discriminating glioblastoma from single brain metastasis. *Acta radiologica (Stockholm, Sweden : 1987)* **60**, 356-366, doi:10.1177/0284185118780889 (2019).

11 Petrujkić, K. *et al.* Computational quantitative MR image features - a potential useful tool in differentiating glioblastoma from solitary brain metastasis. *European journal of radiology* **119**, 108634, doi:10.1016/j.ejrad.2019.08.003 (2019).

12 Mouthuy, N., Cosnard, G., Abarca-Quinones, J. & Michoux, N. Multiparametric magnetic resonance imaging to differentiate high-grade gliomas and brain metastases. *Journal of neuroradiology = Journal de neuroradiologie* **39**, 301-307, doi:10.1016/j.neurad.2011.11.002 (2012).
